# Elevated Plasma Angiopoietin-2 Levels and Primary Graft Dysfunction after Lung Transplantation

**DOI:** 10.1371/journal.pone.0051932

**Published:** 2012-12-19

**Authors:** Joshua M. Diamond, Mary K. Porteous, Edward Cantu, Nuala J. Meyer, Rupal J. Shah, David J. Lederer, Steven M. Kawut, James Lee, Scarlett L. Bellamy, Scott M. Palmer, Vibha N. Lama, Sangeeta M. Bhorade, Maria Crespo, Ejigayehu Demissie, Keith Wille, Jonathan Orens, Pali D. Shah, Ann Weinacker, David Weill, Selim Arcasoy, David S. Wilkes, Lorraine B. Ware, Jason D. Christie, Lung Transplant Outcomes Group

**Affiliations:** 1 Pulmonary, Allergy, and Critical Care Division, Perelman School of Medicine at the University of Pennsylvania, Philadelphia, Pennsylvania, United States of America; 2 Department of Medicine, University of Pennsylvania School of Medicine, Philadelphia, Pennsylvania, United States of America; 3 Division of Cardiovascular Surgery, University of Pennsylvania School of Medicine, Philadelphia, Pennsylvania, United States of America; 4 Division of Pulmonary, Allergy, and Critical Care Medicine, Columbia University College of Physicians and Surgeons, New York, New York, United States of America; 5 Center for Clinical Epidemiology and Biostatistics, University of Pennsylvania School of Medicine, Philadelphia, Pennsylvania, United States of America; 6 Penn Cardiovascular Institute, University of Pennsylvania School of Medicine, Philadelphia, Pennsylvania, United States of America; 7 Division of Pulmonary, Allergy, and Critical Care Medicine, Duke University, Raleigh-Durham, North Carolina, United States of America; 8 Division of Pulmonary, Allergy, and Critical Care Medicine, University of Michigan, Ann Arbor, Michigan, United States of America; 9 Division of Pulmonary and Critical Care Medicine, University of Chicago, Chicago, Illinois, United States of America; 10 Division of Pulmonary, Allergy, and Critical Care, University of Pittsburgh, Pittsburgh, Pennsylvania, United States of America; 11 Division of Pulmonary and Critical Care Medicine, University of Alabama at Birmingham, Birmingham, Alabama, United States of America; 12 Division of Pulmonary, Allergy, and Critical Care Medicine, Department of Medicine, Johns Hopkins University Hospital, Baltimore, Maryland, United States of America; 13 Division of Pulmonary and Critical Care Medicine, Stanford University, Palo Alto, California, United States of America; 14 Division of Pulmonary, Allergy, Critical Care, and Occupational Medicine, Indiana University School of Medicine and Indiana University Health Lung Transplant Program, Indianapolis, Indiana, United States of America; 15 Departments of Medicine and Pathology, Microbiology and Immunology, Vanderbilt University, Nashville, Tennessee, United States of America; Beth Israel Deaconess Medical Center, United States of America

## Abstract

**Introduction:**

Primary graft dysfunction (PGD) is a significant contributor to early morbidity and mortality after lung transplantation. Increased vascular permeability in the allograft has been identified as a possible mechanism leading to PGD. Angiopoietin-2 serves as a partial antagonist to the Tie-2 receptor and induces increased endothelial permeability. We hypothesized that elevated Ang2 levels would be associated with development of PGD.

**Methods:**

We performed a case-control study, nested within the multi-center Lung Transplant Outcomes Group cohort. Plasma angiopoietin-2 levels were measured pre-transplant and 6 and 24 hours post-reperfusion. The primary outcome was development of grade 3 PGD in the first 72 hours. The association of angiopoietin-2 plasma levels and PGD was evaluated using generalized estimating equations (GEE).

**Results:**

There were 40 PGD subjects and 79 non-PGD subjects included for analysis. Twenty-four PGD subjects (40%) and 47 non-PGD subjects (59%) received a transplant for the diagnosis of idiopathic pulmonary fibrosis (IPF). Among all subjects, GEE modeling identified a significant change in angiopoietin-2 level over time in cases compared to controls (p = 0.03). The association between change in angiopoietin-2 level over the perioperative time period was most significant in patients with a pre-operative diagnosis of IPF (p = 0.02); there was no statistically significant correlation between angiopoietin-2 plasma levels and the development of PGD in the subset of patients transplanted for chronic obstructive pulmonary disease (COPD) (p = 0.9).

**Conclusions:**

Angiopoietin-2 levels were significantly associated with the development of PGD after lung transplantation. Further studies examining the regulation of endothelial cell permeability in the pathogenesis of PGD are indicated.

## Introduction

Primary graft dysfunction (PGD), a form of acute lung injury, represents a major cause of early morbidity and mortality after lung transplantation. Thought to be the result of ischemia-reperfusion injury (IRI), PGD affects between 10–30% of lung transplant recipients [1]–[8]. IRI-induced PGD manifests with epithelial cell injury, impairment of fibrinolytic and coagulation cascades, cytokine and chemokine dysregulation, and alteration of vascular permeability [9].

Altered pulmonary vascular permeability is the result of a complex interplay between various regulatory cytokines and growth factors. Previous studies have focused on the relationship between PGD and cytokines known to be involved in regulating vascular permeability. Higher pre-transplant levels of plasma VEGF predicted the development of severe PGD [10]. Elevated gene expression and protein production of endothelin-1, a mediator of vascular permeability, were associated with an increased incidence of PGD [11]–[12]. The detection of these markers of activated endothelium in the setting of PGD suggests that pulmonary vascular disruption may be a critical factor in the development of the syndrome.

The angiopoietins are a group of vascular growth factors involved in angiogenesis, endothelial cell permeability, and regulation of inflammatory cascades [13]. Angiopoietin 1 (Ang1) serves as a constitutive Tie-2 agonist promoting vessel stability and preventing vascular leak. In contrast, Ang2 is abundant in the resting state, but when released by activated endothelium, results in increased endothelial permeability and controls endothelial responses to inflammatory stimuli, including tumor necrosis factor-alpha [14]. Ang2 production is induced by VEGF, hypoxia, and hyperoxia and competes with Ang1, functioning as a Tie-2 receptor antagonist in most contexts [14]. Several studies demonstrate that patients with acute lung injury (ALI) and acute respiratory distress syndrome (ARDS) have higher levels of both plasma and bronchoalveolar lavage Ang2 and that elevated plasma Ang2 levels are associated with increased mortality [15]–[21].

Given the strong association of Ang2 with all-cause ARDS, we hypothesized that lung transplant recipients who developed PGD would have higher post-transplant plasma Ang2 levels compared to those without PGD.

## Materials and Methods

### Ethics Statement

Approval from the Institutional Review Board of each site (University of Pennsylvania, Columbia University, University of Alabama-Birmingham, Johns Hopkins University, Duke University, Indiana University, University of Michigan, University of Pittsburgh, University of Chicago, Vanderbilt University, and Stanford University) and informed consent from each subject were obtained. Consent from all participants was written and the consent form utilized for enrollment was approved by each of the participating center’s Institutional Review Boards listed above. A signed consent form was a requirement for enrollment in the cohort and inclusion in this study. None of the research performed as part of this study was performed outside of the United States.

### Subject Selection

A multicenter, nested case control study was performed on study subjects selected from eight of the eleven participating centers within the ongoing Lung Transplant Outcomes group (LTOG) cohort. Subjects were eligible for inclusion if they underwent lung transplantation between July 2002 and September 2009 for an indication of either IPF or COPD.

Cases were defined by development of any grade 3 PGD within 72 hours of allograft reperfusion. Controls were defined as subjects who did not meet the criteria specified for cases. Cases and controls were frequency matched for predisposing diagnosis leading to transplant. Study subjects were limited to those with IPF or COPD, the two most common indications for lung transplantation, in order to ensure power for a within diagnosis analysis. This study population has been used previously to evaluate the association of leptin and long pentraxin-3 with PGD after lung transplantation [6], [7].

### PGD Grade Determination

The primary case definition for this study was the development of any grade 3 PGD within the first 72 hours after allograft reperfusion. PGD grades were determined according to the International Society for Heart and Lung Transplantation (ISHLT) [22]. Two trained physicians independently assessed chest x-rays from immediately post-transplantation and from 24, 48, and 72 hours after transplantation and assigned PGD grades for each x-ray (subject-level classification kappa = 0.95). Following exclusion of secondary causes, grade 3 PGD was defined by the presence of diffuse alveolar infiltrates and a ratio of the partial pressure of arterial oxygen to the fraction of inspired oxygen (PaO2/FiO2) of less than 200.

### Measurement of Ang2 Concentration

Blood samples were collected from the participants preoperatively and 6 and 24 hours after reperfusion. Plasma samples were processed within 30 minutes and then stored at −80°C. Ang2 plasma level was measured in duplicate utilizing a commercially available ELISA assay (R & D Systems, Minneapolis MN). The mean coefficient of variation for the assay was 2.3%. All study personnel were blinded to the PGD status of the study subjects.

### Statistical Analysis

Study subject characteristics were compared using parametric and non-parametric tests as appropriate. Data were missing from 0–19% of the patient characteristics; mean pulmonary artery pressure was the only covariate with more than 10% missingness. Simple imputation was used to account for missing covariate data. The primary analysis utilized generalized estimating equations (GEE) to identify differences in Ang2 levels over time and across study subjects. Based on previous studies identifying differences in biomarker levels across predisposing diagnoses, we a priori defined diagnosis leading to transplantation as a possible effect modifier and performed diagnosis specific analyses [5], [6]. Potential confounding by recipient and donor age, sex, and race/ethnicity, cardiopulmonary bypass use, transplant surgical type, ischemic time, intra-operative mean pulmonary artery pressure, and packed red blood cell (PRBC) transfusion volumes was assessed individually using multivariable GEE modeling. A change in the β coefficient, after inclusion of a covariate, of greater than 20% was used to identify confounding [23]. A sensitivity analysis was performed using the presence of grade 3 PGD at 72 hours after reperfusion as a more severe phenotype. The secondary analysis was a comparison of median Ang2 levels prior to transplant and 6 and 24 hours after transplant using Wilcoxon rank sum tests across groups. A p<0.05 was pre-defined for statistical significance for all tests. All statistical analyses were performed using Stata 11.2 software (STATA Corp., College Station, TX).

## Results

Forty cases and 79 controls were included for analysis. Donor, recipient and intra-operative characteristics are presented in Table 1. Cases and controls demonstrated similar donor and recipient characteristics. There was a higher incidence of cardiopulmonary bypass use among cases compared with controls (54% vs. 30%, p = 0.01). PGD cases received a larger volume of packed red blood cells intraoperatively than controls (1063 ml vs. 696 ml, p = 0.04). There were 16 cases with COPD and 24 cases with IPF.

**Table 1:** Subject characteristics stratified as PGD cases and non-PGD controls.

Characteristics	PGD (n = 40)	Non-PGD (n = 79)	p
Recipient			
Age, yr	55 (52, 58)	56 (53, 58)	0.7
Female Gender	30%	46%	0.1
Race			0.1
Caucasian	80%	90%	
African American	13%	5%	
Hispanic	0%	4%	
Asian	5%	1%	
Other	3%	0%	
Donor			
Age, yr	34 (29, 38)	33 (30, 36)	0.7
Female Gender	48%	43%	0.6
Race			0.2
Caucasian	73%	62%	
African American	15%	22%	
Hispanic	13%	9%	
Asian	0%	6%	
Other	0%	1%	
Cause of death			0.9
Blunt Trauma	3%	4%	
Head Trauma	30%	35%	
Suicide	3%	0%	
Stroke	48%	34%	
Anoxia	0%	11%	
Other	18%	15%	
Recipient Diagnosis			0.9
COPD	40%	41%	
IPF	60%	59%	
Transplant Type, single	38%	38%	0.9
Use of Cardiopulmonary Bypass	54%	30%	0.01
Time on Bypass, min	235 (201, 270)	210 (184, 236)	0.2
Ischemic Time, min	308 (281, 334)	282 (262, 302)	0.1
Mean Pulmonary Artery Pressure, mmHg	47 (40, 54)	41 (36, 46)	0.3
Packed Red Blood Cells, ml	1063 (675, 1450)	696 (538, 854)	0.04
Fresh Frozen Plasma, ml	893 (702, 1084)	1062 (769, 1354)	0.3
Platelets, ml	421 (99, 743)	228 (46, 411)	0.3

PGD is defined as any grade 3 PGD during first 72 hours.

Continuous variables are expressed as means with 95% confidence intervals, while dichotomous and categorical variables are expressed as percentages, which may not exactly total 100% because of rounding.

**Figure 1:**
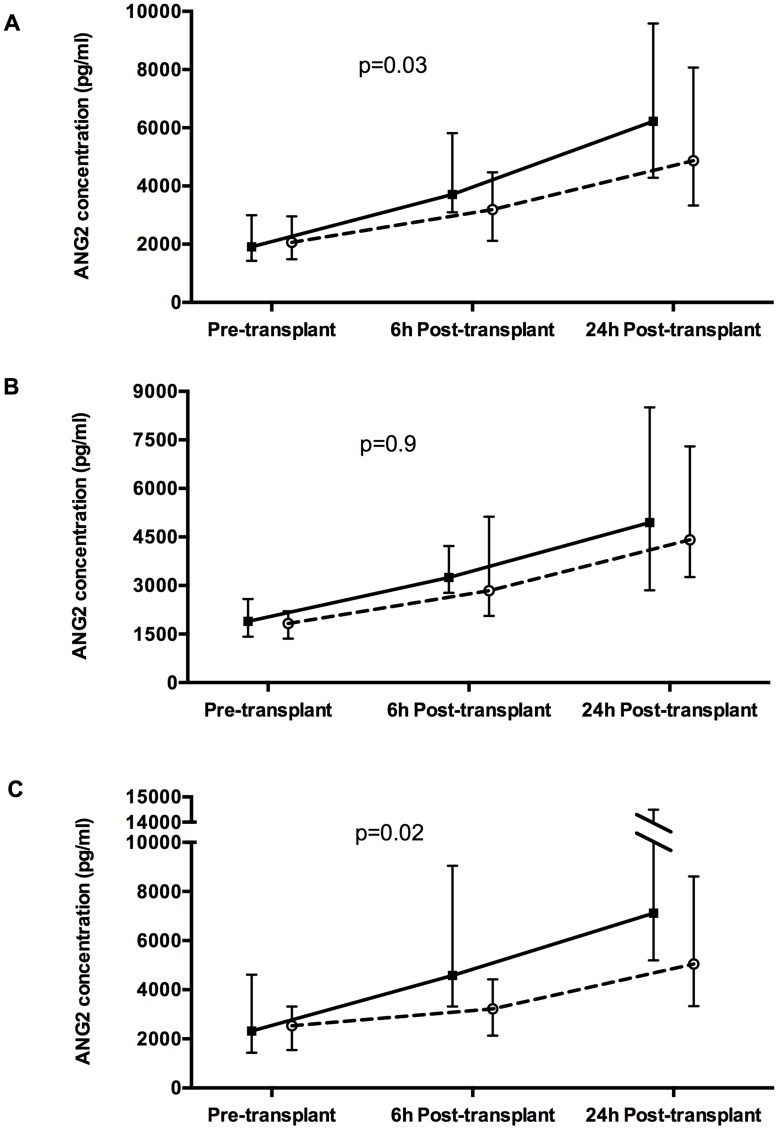
Longitudinal median Angiopoietin 2 level across the pre-transplant, 6-h post-transplant, and 24-h post-transplant time points. A) all study subjects, B) subjects with COPD, and C) subjects with IPF. Solid line represents PGD cases and dashed line represents PGD-free controls. Error bars represent the 95% CI. All p-values reported are from GEE modeling.

GEE models were used to assess the association between change in Ang2 level over time and the risk of PGD (Figure 1). While Ang2 levels increased from pre-transplant to post-transplant measurements in all subjects, there were significant differences in the slope of the change in Ang2 level over time in cases compared to controls (p = 0.03). Among subjects limited to a diagnosis of COPD, there were no significant differences in longitudinal Ang2 levels between cases and controls (p = 0.9). In those subjects transplanted for IPF, GEE longitudinal modeling identified a significant difference in the trend of Ang2 levels across all three time points between cases and controls, with cases having significantly larger increases in Ang2 plasma levels compared with controls (p = 0.02).

In the overall study population, there was no significant difference in median Ang2 concentrations between cases and controls prior to transplantation (1909 pg/ml vs. 2064 pg/ml; p = 0.9) or 24 hours after allograft reperfusion (6221 pg/ml vs. 4869 pg/ml; p = 0.1), with borderline significance at 6 hours (3707 pg/ml vs. 3188 pg/ml; p = 0.05). Pre-transplant levels of Ang2 were significantly higher in patients with IPF vs. COPD (3330 pg/ml vs. 2051 pg/ml, p = 0.005). Among patients with COPD, there was no significant difference in Ang2 level between cases and controls pre-transplant (1895 pg/ml vs. 1824 pg/ml), 6 hours after transplant (3247 pg/ml vs. 2843 pg/ml) or 24 hours after transplant (4944 pg/ml vs. 4412 pg/ml) (p>0.6 for all time points). Among patients with IPF, Ang2 concentrations at 6 hours were significantly higher in cases compared to controls (4578 pg/ml vs. 3218 pg/ml, p = 0.01) with borderline significance at 24 hours (7115 pg/ml vs. 5046 pg/ml, p = 0.06). There was no significant difference in Ang2 level prior to transplant in the IPF cases and controls (2538 pg/ml vs. 2318 pg/ml, p = 0.6).

The effects of potential confounders on Ang2 levels and the development of PGD were assessed using multivariable GEE modeling (Table 2). The use of cardiopulmonary bypass and mean pulmonary artery pressure confounded the association between longitudinal Ang2 level and PGD, resulting in loss of statistical significance on adjustment. There was no evidence of confounding of the relationship between PGD and Ang2 level by any donor or recipient characteristics or by total ischemic time, blood product transfusion volume, or transplant type (Table 2).

**Table 2:** Generalized estimating equation (GEE) analysis of association between PGD and Ang2 plasma level.

Model	GEE β-coefficient for PGD, ×10^−5^(95%CI)	p-value
Unadjusted Base Model (n = 119)	5.6 (0.5, 10.8)	0.03
COPD only (n = 48)	−1.0 (−13, 11)	0.9
IPF only (n = 71)	7.6 (1.4, 13.8)	0.02
Base Model Adjusted for (n = 119):		
Cardiopulmonary Bypass	3.5 (−1.9, 8.8)	0.2
Transplant Type	5.8 (0.5, 11.1)	0.03
Recipient Age	5.6 (0.4, 10.7)	0.03
Recipient Sex	6.4 (1.0, 11.9)	0.02
Donor Age	5.7 (0.5, 10.9)	0.03
Donor Sex	5.5 (0.4, 10.7)	0.04
Total ischemic time	4.5 (−0.7, 9.6)	0.09
Mean Pulmonary Artery Pressure	4.2 (−1.4, 9.7)	0.1
Packed Red Blood Cells	4.7 (−0.6, 10.0)	0.08

β-coefficient and p-value are generated from GEE models.

COPD = Chronic Obstructive Pulmonary Disease.

IPF = Idiopathic Pulmonary Fibrosis.

A sensitivity analysis performed defining cases as those subjects with grade 3 PGD at 72 hours produced similar results. GEE modeling among all study subjects identified a significant difference in the change in Ang2 level over time in cases compared to controls (p = 0.04). Among COPD subjects, there was no significant association between Ang2 trend and the development of PGD (p = 0.9). Among IPF subjects, there was a significant difference in the change in Ang2 level across the three time points between cases and controls (p = 0.02).

## Discussion

The purpose of this study was to define the association of plasma Ang2 concentrations with the development of PGD after lung transplantation. We found that post-transplant Ang2 plasma levels were significantly associated with the development of PGD. There was also a significant difference in the change in Ang2 concentration over time between patients with IPF who developed PGD compared with those without PGD.

Our identification of an association of elevated Ang2 levels and PGD is consistent with evidence for a central role of Ang2 in the development of syndromes with similar mechanisms to PGD. Variation in the ANGPT2 gene, which encodes Ang2, was associated with risk of trauma-associated ALI and with a higher proportion of full-length Ang2 plasma isoforms [16]. Circulating levels of Ang2 are also significantly higher in trauma patients with ALI compared to matched ALI-free controls [24]. Ang2 levels were also significantly higher in patients with severe sepsis compared to those with sepsis alone or to those without sepsis or systemic inflammatory response syndrome [20]. Higher Ang2 plasma levels were associated with increased mortality in a surgical population with ALI and in the NHLBI ARDS network population. Mechanistically, both exogenous Ang2 or plasma from patients with ALI induces permeability in vitro, and normal barrier function can be restored by adding Ang1 [15]. Experimental models to augment Ang1 with either cell-based or mesenchymal stem cell therapy resulted in mitigated parameters of lung injury [25], [26]. Given the shared pathology between ALI and PGD, our findings are consonant with studies of Ang2’s role in ALI. We now extend the relevance of Ang2 to the lung transplant population.

The results of this study provide further evidence for a significant role of endothelial barrier disruption in the development of PGD. Krenn et al. have demonstrated that VEGF levels are higher in patients with cystic fibrosis (CF) compared to those with COPD and that CF patients are at higher risk of PGD compared to those with COPD. Furthermore, CF patients had higher lung water content post-reperfusion compared to COPD subjects [27]. In a general population of lung transplant recipients, pre-transplant VEGF serum concentrations were higher in patients ultimately developing PGD compared to those without PGD [10]. The consistent demonstration of an association between upregulated Ang2 production across related syndromes, combined with the strong association between endothelial disruption and the development of PGD, adds validity to our identification of an association between post-transplant Ang2 plasma levels and PGD.

There was evidence of confounding of the relationship between plasma Ang2 level and the development of PGD by cardiopulmonary bypass and pulmonary arterial pressures. Elevated pulmonary pressures and bypass are both independent risk factors for PGD [28]–[30]. Bypass use has been associated with elevations in Ang2 plasma levels and increased vascular permeability [31], [32]. Pulmonary arterial hypertension has also been associated with elevated plasma Ang2 levels with increased Ang2 gene expression in plexiform lesions from lung tissue samples [33]. Abnormalities in the Ang2/Tie2 pathway may in part explain the association of bypass and elevated pulmonary pressures with PGD; that is, longer cardiopulmonary bypass time may induce Ang2 release from endothelium, triggering vascular barrier breakdown and fluid extravasation, therefore contributing pathogenically to PGD.

A subgroup analysis stratified by pre-operative diagnosis identified a significant association between plasma Ang2 levels and PGD in the IPF subgroup, but not the COPD subgroup. Furthermore, the association between change in Ang2 level over the peri-operative time period and PGD was only significant in the IPF subgroup, suggesting that the role of Ang2 in the development of PGD is diagnosis-specific. We have identified significant differences in the association of plasma levels of long pentraxin-3, an innate immune mediator, and clara cell secretory protein, a marker of epithelial cell disruption, and PGD when comparing patients with fibrotic and non-fibrotic lung disease [5], [6]. Ang2 is overexpressed and Ang1 expression decreased in patients with IPF compared to those with connective tissue disease associated pulmonary fibrosis, indicating an altered angiogenic profile in patients with IPF [34]. Likewise, prior studies have demonstrated that serum VEGF levels were significantly higher in patients with CF compared to COPD, both before transplant and immediately after allograft reperfusion [27]. The diagnosis-specific association of Ang2 plasma levels with PGD provides further evidence that PGD may have differential involvement of cellular pathways depending on the pre-operative pulmonary diagnosis.

There are several limitations to this study. While we hypothesize that the association of elevated Ang2 plasma levels and PGD may indicate disruption of pulmonary endothelial cells with leakage into the systemic circulation, we cannot be sure of the source of the Ang2 release into plasma. While Ang2 is generally believed to be predominantly an endothelial protein resident in Weibel-Palade bodies, it has been demonstrated in alveolar epithelial cells as well [18]. In the absence of lung biopsy samples with staining for Ang2 localization, we cannot determine the cellular source of the Ang2 detected in plasma. Additionally, systemic endothelium may be the source of the Ang2. The functional implications of the association between PGD and elevated plasma Ang2 levels are an area for future study. We were unable to evaluate the association between donor Ang2 levels at the time of organ procurement and the development of PGD in the recipient. With the increased utilization of *ex* vivo lung perfusion (EVLP) to improve allograft function, changes in Ang2 levels from the allograft prior to implantation may also correlate with PGD risk. EVLP may be a powerful tool to analyze the role of the Ang2 pathway in the pathogenesis of PGD. There is also the potential for PGD misclassification. Evaluation of chest x-rays for the development of PGD was performed by two independent physicians and grade was assigned using a validated scoring system [22], [35]. Sensitivity analysis using a more severe phenotype of PGD, grade 3 at 72 hours after reperfusion, confirmed our findings. Our evaluation of the Tie-2/Ang2 pathway is limited to plasma Ang2 levels. Recent studies have demonstrated utility in the Ang2/Ang1 ratio for predicting poor outcomes associated with ALI, and genetically-determined splice variants have been identified by our group [16], [36]. Plasma samples for the patients included in this study have been exhausted, limiting our ability to further analyze the role of Ang2/Ang1 ratio, different isoforms, or the role of VEGF plasma level in PGD and will be the focus of future investigations. While the Ang-2/Ang-1 ratio may be considered a measure of relative antagonism and agonism of the Tie-2 receptor as hypothesized by Ong et al., it is not clear that the ratio adds to the association demonstrated between Ang2 alone and PGD [36]. Tie2 phosphorylation studies are beyond the scope of this study as no biopsy samples are taken as part of the LTOG protocol, either prior to organ procurement or after allograft implantation and reperfusion. Finally, as with previous biomarker evaluations of PGD, given the lack of significant pre-transplant differences in Ang2 plasma levels between cases and controls, it is not possible to define a causal relationship between Ang2 production and the development of PGD post-transplant. Ang2 measurements were in some cases made concurrently with the identification of PGD. In some cases, chest radiographs and oxygenation parameters demonstrated a patient had PGD prior to the 6 and 24 hour collection times used in this study. Therefore, Ang2 plasma level does not serve as a useful diagnostic tool for PGD. However, we can conclude a likely mechanistic role for endothelial disruption and the development of PGD, and that the Tie2/Ang2 pathway may be an ideal target for pharmacologic intervention.

In summary, we identified elevated Ang2 plasma levels to be strongly associated with the development of PGD after lung transplantation for patients with IPF. This provides increasing support for a central role for endothelial cell disruption in the development of PGD. Evaluating the role for potential therapeutics targeting aberrant vascular permeability, including EVLP is an important area of future study.
